# Interbreeding between local and translocated populations of a cleaner fish in an experimental mesocosm predicts risk of disrupted local adaptation

**DOI:** 10.1002/ece3.5246

**Published:** 2019-05-09

**Authors:** Enrique Blanco Gonzalez, Sigurd H. Espeland, Sissel Jentoft, Michael M. Hansen, Joana I. Robalo, Nils C. Stenseth, Per Erik Jorde

**Affiliations:** ^1^ Department of Natural Sciences University of Agder Kristiansand Norway; ^2^ Centre for Coastal Research University of Agder Kristiansand Norway; ^3^ Norwegian College of Fishery Science UiT The Arctic University of Norway Tromsø Norway; ^4^ Institute of Marine Research Flødevigen Norway; ^5^ Centre for Ecological and Evolutionary Synthesis, Department of Biosciences University of Oslo Oslo Norway; ^6^ Department of Bioscience Aarhus University Aarhus C Denmark; ^7^ MARE ‐ Marine and Environmental Sciences Centre ISPA Instituto Universitário de Ciências Psicológicas, Sociais e da Vida Lisboa Portugal

**Keywords:** corkwing wrasse, mating behavior, microsatellites, parentage assignment, reproductive fitness, *Symphodus melops*

## Abstract

Translocation of organisms within or outside its native range carries the risk of modifying the community of the recipient ecosystems and induces gene flow between locally adapted populations or closely related species. In this study, we evaluated the genetic consequences of large‐scale translocation of cleaner wrasses that has become a common practice within the salmon aquaculture industry in northern Europe to combat sea lice infestation. A major concern with this practice is the potential for hybridization of escaped organisms with the local, recipient wrasse population, and thus potentially introduce exogenous alleles and breaking down coadapted gene complexes in local populations. We investigated the potential threat for such genetic introgressions in a large seminatural mesocosm basin. The experimental setting represented a simulated translocation of corkwing wrasse (*Symphodus melops*) that occurs on a large scale in the Norwegian salmon industry. Parentage assignment analysis of mesocosm's offspring revealed 30% (195 out of 651 offspring) interbreeding between the two populations, despite their being genetically (*F*_ST_ = 0.094, *p* < 0.05) and phenotypically differentiated. Moreover, our results suggest that reproductive fitness of the translocated western population doubled that of the local southern population. Our results confirm that human translocations may overcome the impediments imposed by natural habitat discontinuities and urge for immediate action to manage the genetic resources of these small benthic wrasses.

## 1 INTRODUCTION

Organisms are frequently released into natural environments intentionally or inadvertently, such as via ship's ballast water or escapees from aquaculture facilities (Laikre, Schwartz, Waples, & Ryman, 2010; Swan, McPherson, Seddon, & Moehrenschlager, 2016). Human‐mediated translocations have been reported for a wide range of taxa including seaweeds, invertebrates, and vertebrates (see reviews by Geller, Darling, & Carlton, 2010, Thomas, 2011). Despite its potential for species conservation, the risk of becoming invasive, as well as the ecological and genetic interactions between native and exogenous populations, remains a common topic of debate for managers and conservationists (Araki & Schmidt, 2010; Laikre et al., 2010; Thomas, 2011). Putative deleterious genetic risks, associated with large‐scale releases and translocations, include the loss of genetic variation and adaptations as well as alterations in genetic profiles and population structure (Geller et al., 2010; Hӓnfling, 2007; Laikre et al., 2010).

Experimental studies assessing reproductive fitness between exogenous and locally adapted populations have drawn contradictory results. While many studies suggested lower fitness performance in nonlocal (hatchery‐released, unintentionally escaped and translocated) organisms (Araki, Cooper, & Blouin, 2009; Christie, Ford, & Blouin, 2014; Eldridge & Naish, 2007; Glover et al., 2017), others have reported no fitness disadvantage (Berejikian, Van Doornik, Scheurer, & Bush, 2009; Blanco Gonzalez, Nagasawa, & Umino, 2008; Delgado & Glazer, 2007; Hess et al., 2012). Pedigree reconstruction in offspring for stocking has often revealed significant variation in the number of parental contributors and their family sizes, and consequently, in their effective population sizes and rate of inbreeding (Blanco Gonzalez, Taniguchi, & Umino, 2010; Jeong, Blanco Gonzalez, Morishima, Arai, & Umino, 2007). Currently, our knowledge on reproductive fitness of nonlocal populations is highly biased toward studies conducted on salmonids, with very little information on strictly marine fish species (Araki & Schmidt, 2010). The large population sizes and extensive geographic areas commonly occupied by most marine fish make these studies very challenging (see references in Araki & Schmidt, 2010; Blanco Gonzalez, Aritaki, Knutsen, & Taniguchi, 2015). Large‐scale mesocosm facilities may offer an alternative to overcome these limitations (Blanco Gonzalez et al., 2010; Jeong et al., 2007). Although such seminatural experimental setup may not fully resemble processes taking place in the wild, it will at least provide a good indication of the spawning performance as compared to laboratory culture conditions (Leggatt et al., 2014).

Sea lice infestation is a major burden for the aquaculture industry causing high salmon mortalities and large economic losses (Blanco Gonzalez & de Boer, 2017; Iversen, 2016). The use of wrasses as cleaner fish has been proposed to be the most economical and environmental friendly solution to combat sea lice infestation (Liu & Bjelland, 2014), despite high mortalities at low temperatures (Bjelland, Simensen, & Kvenseth, 1996; Costello, 1991; Sayer, Reader, & Davenport, 1996). As result, translocation practices have been undertaken in several European countries, where wild wrasses are sex‐ and size‐selectively fished and transported alive from areas of high abundance to salmon farms located in areas where local supply cannot cope with their high demand (Blanco Gonzalez & de Boer, 2017; Riley, Jeffery, Cochrane‐dyet, White, & Ellis, 2017). In the UK, for example, up to one million wrasses, mainly ballan wrasse *Labrus bergylta*, are translocated from the southwest coast to Scottish farms annually (Riley et al., 2017). In Norway, the largest farmed salmon producer in the world, several millions of wild‐caught adult wrasses, mainly goldsinny *Ctenolabrus rupestris* and corkwing wrasse *Symphodus melops*, from southern regions of Norway and Sweden are translocated to salmon farms located further north in the west coast (Blanco Gonzalez & de Boer, 2017). Several cleaner wrasse species display a strong regional and latitudinal variation in sexual size dimorphism (Halvorsen et al., 2016; Sayer, Gibson, & Atkinson, 1996). Human‐mediated translocation may facilitate crossing the geographical boundaries delimited by natural barriers and evolutionary processes (Geller et al., 2010) and could therefore pose a major threat to local wrasse populations if a significant number of wrasses are released from the net pens unintentionally or intentionally (Espeland et al., 2010).

Recent genetic studies of cleaner fishes in northern Europe have revealed marked large‐scale population genetic structure, with reduced genetic variability in northern populations (Almada et al., 2017; Blanco Gonzalez, Knutsen, & Jorde, 2016; Jansson et al., 2017; Knutsen et al., 2013; Robalo et al., 2012). The northern Scandinavian populations have shown a further pattern of isolation by distance in the goldsinny (Jansson et al., 2017) and a genetic discontinuity (“break”) between southern and western Norwegian populations in the case of corkwing wrasse (Blanco Gonzalez et al., 2016). Moreover, apparent translocated individuals of goldsinny (Jansson et al., 2017) and corkwing wrasse (Faust, Halvorsen, Andersen, Knutsen, & André, 2018) have been found in the proximities of salmon farms in Norway, with indication of hybridization with locals for the latter species. Little is known about how translocated individuals fare in their new environment, their mating preferences versus local conspecifics, and potential isolation mechanisms between translocated and local wrasse populations. Hence, the risk of spreading of exogenous genes in these cleaner fishes is presently unclear, despite their large‐scale deployment in the industry.

In the present study, we addressed several critical questions concerning the reproductive behavior of translocated fish: (a) Do local and translocated wild‐caught marine fish interbreed? (b) Do locally adapted populations present reproductive fitness advantage over translocated individuals? (c) Do specimens display any mating preference regarding origin or phenotypic traits?

## 2 MATERIAL AND METHODS

### 2.1 Study species and experimental setting

Rising sea water temperatures registered in the last decades have favored the increase in abundance of corkwing wrasse in Norway (Barceló, Ciannelli, Olsen, Johannesen, & Knutsen, 2016; Knutsen et al., 2013). The species is nest‐building and displays alternative reproductive tactics (Halvorsen et al., 2016; Sayer, Gibson, et al., 1996). Large nesting males build and defend nests from sneaker males (Potts, 1984). These different life‐history tactics are particularly pronounced in the west coast of the Scandinavian Peninsula where nesting males show a delay in maturation time and faster growth rates compared to females and sneaker males (Halvorsen et al., 2016).

The present experimental study was conducted at the research facilities of the Institute of Marine Research at Flødevigen in Arendal, on the south coast of Norway. The mesocosm basin has a capacity of approximately 2,000 m^3^, a surface area of 660 m^2^, a maximum depth of 5 m, and the seawater is pumped up from 75 m depth (Moksness, 1982). The geographical location and suitability of these facilities for the study justify moving specimens from the west coast to the south, instead of in the opposite direction which is the one most commonly used by the salmon industry (Blanco Gonzalez & de Boer, 2017). On June 24, 2014, 167 adults from Norheimsund, on the west coast of Norway (for location in a map see Blanco Gonzalez et al., 2016), were collected using baited wrasse pots by a local fisherman and transported alive to Flødevigen. After arrival, all individuals were measured in total length (cm), weighted (g; see Table 1 for details on wrasse samples). The sex of every adult was determined by examination of the urogenital papilla (only present in females and sneaker males but not in nesting males) and inspecting an ejaculate sample of egg/sperm obtained by applying gentle pressure on the abdomen. Following these procedure, we confirmed that all individuals were sexually mature, and they were classified as nesting male, sneaker male, and female. In addition, a small piece of the tail was clipped and stored in 95% ethanol for DNA analysis before they were released into the mesocosm basin. These individuals represent the group of translocated specimens of nonlocal west origin. On July 1, 2014, a second group of 151 adult fish were collected from Arendal, close to Flødevigen, using baited wrasse pots by a local fisherman and transported to Flødevigen. They were subjected to the same measurements and handling as the earlier sample, except that the ventral side of their bodies was tagged with pink visible implanted elastomer. These specimens comprise the group of breeders of local south origin of the study and were released into the mesocosm basin together with the western group. The two group of wrasses were maintained at the mesocosm for a full year and allowed to spawn naturally during the upcoming spawning season in spring–summer. At the end of the spawning season, between July 30 and August 19, 2015, a total of 651 offspring were collected with small aquarium nets along the edges of the spawning basin. Offspring were weighted (g) and measured in total length (cm) after the image analysis of the digital photography taken with a Tucsen CMOS IS1000 camera (Tucsen) attached to a Leica MZ16a stereomicroscope (Leica). A small piece of the juvenile's tail was clipped and stored in 95% ethanol for DNA analysis.

**Table 1:** Summary of corkwing wrasse samples analyzed in this study

Sample ID	Origin	Collection date	Tag color	Sex	Sample size	Total length range (mean ± *SD*)	Body weight range (mean ± sd)
Breeder south	Wild	24.06.2014	Yellow	Male	64	10.5–20.5 (14.0 ± 2.6)	16.2–108 (39.8 ± 22.6)
				Sneaker	9	11.5–12.5 (11.8 ± 0.3)	18.2–27 (21.1 ± 2.6)
				Female	76	10.5–20 (14.4 ± 2.4)	14.6–103.8 (44.5 ± 22.0)
Breeder west	Wild	01.07.2014	Pink	Male	59	10.5–17.0 (12.8 ± 1.3)	16.4–59.3 (30.1 ± 8.6)
				Sneaker	24	10.5–14.5 (12.3 ± 0.9)	17.1–37.2 (25.7 ± 5.0)
				Female	86	10.5–19.5 (12.9 ± 1.3)	16.5–101.7 (30.0 ± 11.2)
Offspring	Mesocosm	30.07–19.08.2015		Immature	651	0.4–3.5 (1.7 ± 0.5)	0.8–0.1 (0.1 ± 0.1)

Note

Sample ID, origin, collection date, tag color, sex, sample size, total length range (mean ± standard deviation, *SD*) in cm, and body weight range (mean ± standard deviation, *SD*) in g.

### 2.2 Microsatellite genotyping

Total genomic DNA was extracted from ethanol‐preserved tail samples using E.Z.N.A® Tissue DNA kit (Omega Bio‐Tek), resuspending the DNA in TE buffer. The ability of microsatellite loci to resolve parentage assignment depends on their number as well as their degree of polymorphism (Villanueva, Verspoor, & Visscher, 2002). The analysis was conducted on eleven polymorphic microsatellites characterized in previous studies on corkwing wrasse: SMD121, SMA11, SMA103, SMD131, SMD110, SMD112, SMB11, SMC8, SMB101, SMC5, SMD118, SMB101, SMC5, and SMD118, following the same multiplex PCR protocols and dye labeling as previously described (Blanco Gonzalez et al., 2016; Knutsen et al., 2013; Knutsen & Sannæs, 2009). PCR amplifications were carried out in a multiplex reaction of 10 μl volume including 10 pmol of each primer and 1 μl of template DNA, corresponding to 30–50 ng. PCR conditions for the multiplex reaction of three new primers consisted of an initial denaturation step at 94°C for 5 min, followed by 35 cycles of 95°C for 30 s, annealing at 56°C for 60 s and 72°C for 60 s, with a final extension at 72°C for 15 min. One microliter of PCR product was mixed with 10 µl of Hi‐Di formamide and 0.8 µl of GeneScan^Tm^—600 Liz (Applied Biosystems) and run on an ABI 3130XL automated sequencer. Individual genotypes were assessed with GENEMAPPER v. 4.0 (Applied Biosystems). As a guard against potential genotyping errors, all samples were run with the same size standard and on the same machine. In addition, approximately 5% of the samples were randomly subjected to repeated genotyping.

### 2.3 Genetic diversity and differentiation

Genetic variation was assessed by counting observed alleles (*A*), and calculating allelic richness (*A*_r_), observed (*H*_O_), and expected heterozygosity (*H*_E_), both within samples and for the total over all samples (*H*_T_), based on Nei and Chesser (1983), using FSTAT v.2.9.3.2. (Goudet, 1995) and the divBasic function in diveRsity package v.1.9.90 (Keenan, McGinnity, Cross, Crozier, & Prodöhl, 2013) in R (R Development Core Team, 2011). GENEPOP package v.4.7.0. (Rousset, 2008) in R was used to estimate the inbreeding coefficient *F*_IS _(Weir & Cockerham, 1984) and to test for deviations from Hardy–Weinberg (HW) equilibrium by the Markov chain procedure with 100,000 demorization steps, 1,000 batches, and 50,000 iterations per batch. The false discovery rate (FDR) approach (Benjamini & Hochberg, 1995) was adopted when interpreting the significance of *p* values in situations of multiple tests. GENEPOP v.4.7.0 was also used with the same Markov chain parameters to test for linkage disequilibrium (LD) among all pairs of loci for each sample. MICROCHECKER v.2.2.1 (Van Oosterhout, Hutchinson, Wills, & Shipley, 2004) was employed to investigate the presence of null alleles, stuttering errors, or technical artifacts. The presence of null alleles was further assessed by estimating null allele frequencies (fn) with CERVUS v.3.0 (Kalinowski, Taper, & Marshall, 2007).

The discrimination power of the set of microsatellite loci for parentage analysis was determined by the polymorphic information content (PIC) and the exclusion probability (*Q*) with CERVUS v.3.0 (Kalinowski et al., 2007). We also used GenAlEx (Peakall & Smouse, 2012) to estimate the probability of identity index (*I*), an index representing the probability of finding two individuals sharing a multilocus genotype. In addition, we determined the cumulative success rate of parentage allocation ranking the markers according to the exclusion probability of both parents, Excl. P2 option, based on 1,000 offspring simulations using the PFX_Mchoice macro implemented in PARFEX v1.0 (Sekino and Kakehi 2011).

Genetic differentiation among samples was conducted by Wright's *F*_ST_, using Weir and Cockerham's (1984) estimator *θ* applied to all samples and between pairs of samples. The statistical significance of *p *values was examined by *G *tests in GENEPOP package v.4.7.0. (Rousset, 2008) with 100,000 demorization steps, 1,000 batches, and 50,000 iterations per batch. The FDR approach proposed by Benjamini and Yukutieli (2001) was adopted to correct for multiple tests in pairwise tables.

### 2.4 Pedigree reconstruction and effective number of breeders

Parentage assignment was performed in PAPA v.2.0 (Duchesne, Godbout, & Bernatchez, 2002) and corroborated in CERVUS v.3.0 (Kalinowski et al., 2007). Pedigree reconstruction in PAPA v.2.0 requires a closed system where all putative breeders have been sampled (Duchesne et al., 2002) as was the case in the present study. The analysis was performed with a uniform error of 0.02 on all loci. The assignment analysis in CERVUS is based on the log‐likelihood (LOD) score, inferring parental pairs to those breeders with the highest likelihood. The analysis was conducted considering known broodstock sexes. Allocation was considered correct only when trio (offspring and a parental pair) showed no mismatch at any locus, and PAPA and CERVUS showed consistent parental pair assignments. In both programs, prior to offspring assignment, parentage estimates were simulated for 10,000 offspring. In order to minimize any genotyping errors and ensure a reliable parental‐offspring assignment, we examined those offspring not allocated to any parental pair and those allocated to more than one pair (called as “ambiguous” in PAPA). A new assignment test was performed allowing a maximum mismatch at two loci. Allele scoring for trios was revised at all loci and corrected accordingly. Parental assignment of the few remaining offspring was successfully resolved by repeated genotyping at all loci and re‐running the analysis. Based on these results, offspring were classified as “south” when both parents were of south origin, “west” when both parents were from the west coast, or “hybrid” when parents were of different origins.

Once all parental pairs were identified, we estimated the inbreeding effective number of breeders, Nb (Araki, Waples, Ardren, Cooper, & Blouin, 2007; Crow & Kimura, 1970; Waples, 2002), with and without taking parental origin into consideration. This was calculated from the Nb (*N*), the average number of offspring ($\overset{`}{k}$), and the variance in the number of offspring (*V*_k_) from contributing breeders, as assessed from the parental assignments (above):

$$Nb_{f,m}=\frac{\overset{`}{k}N-2}{\overset{`}{k}-1+\frac{\mathrm{Vk}}{\overset{`}{k}}}.$$

This was calculated separately for male (m) and female (f) parents and combined to obtain the inbreeding effective Nb:

$$\text{Nb}=\frac{4*{\text{Nb}}_{\text{f}}*{\text{Nb}}_{\text{m}}}{{\text{Nb}}_{\text{f}}+{\text{Nb}}_{\text{m}}}.$$

We further investigated whether the presence of sneaker males would contribute to increase Nb or not. The analysis was performed by comparing the estimates when offspring from all breeding pairs were included (regardless nesting or sneaking male behavior) to those obtained excluding the offspring produced by sneaker males.

### 2.5 Assortative mating and reproductive fitness

We used parentage assignment results to test the hypothesis of nonrandom mating between south and west breeders using a 2 × 2 contingency table with Yates correction. The analysis was performed taking into account parental origin (south and west) and sex (female and male). In this analysis, nesting and sneaker males were pooled together due to the small number of sneakers in the samples (see Table 1 for details).

Results of the parentage assignment test were also used to analyze causes of variance in reproductive fitness. For each possible breeding pair, we considered whether offspring were produced or not, parental origin (south and west), sex (nesting males, females and sneaker males), and whether both parents were from same origin or not. Furthermore, we also considered the effects of parental weight/length ratio (both for males and females) to analyze reproduction success. Since most possible male–female pair combinations did not produce offspring (243 identified successful parental pairs out of 25,272 possible breeding pair combinations from 162 females and 156 males, Table 1), we constructed a statistical model where the success/failure (producing offspring or not) was analyzed based on all possible breeding pairs, and another model where the number of offspring was analyzed based only on successful breeding pairs.

The analysis of success/failure was analyzed using a logistic regression model in R (R Development Core Team, 2011):

(1) $$log\left(\frac{p_{\mathrm{ij}}}{1-p_{\mathrm{ij}}}\right)=\beta_{0}+\beta_{1}O_{i}+\beta_{2}O_{j}+\beta_{3}O_{\mathrm{ij}}+\beta_{4}L_{i}+\beta_{5}L_{j}+\beta_{6}S_{i}+\varepsilon_{\mathrm{ij}}.$$

In the formula above, *p_ij_* is the probability that the breeding pair consisting of the *i*'th male and the *j*'th female will produce any offspring. *β* are the regression coefficients or effects of the respective variables. *O_i_* and *O_j_* represent male and female parents' origins and take on a value of 1 if the *i*'th male is from western Norway and 0 if it is from southern Norway and similar for the *j*'th female. *O_ij_* represents the case when parents have the same origin, in which case has the value of 1, or not (value 0). The variables *L_i_* and *L_j_* are the weight/length ratio of the *i*th male and *j*th female, respectively. The variable *S_i_* is a factor variable considering the effect of sneaker behavior on the mating success and takes on a value of 1 if the *i*'th male is a sneaker and 0 if it is a nesting male. The *ε_ij_* is an independent, identical distributed error term for each breeding pair of the i'th male and *j*'th female. The coefficients were estimated using maximum likelihood estimation.

For those breeding pairs that actually produced at least one sampled offspring, we further analyzed the number of offspring in the sample, by using a general linear model with a poison distributed response variable:

(2) $$log\left(N_{\mathrm{ij}}\right)=\gamma_{0}+\gamma_{1}O_{i}+\gamma_{2}O_{j}+\gamma_{3}O_{\mathrm{ij}}+\gamma_{4}L_{i}+\gamma_{5}L_{j}+\gamma_{6}S_{i}+\varepsilon_{\mathrm{ij}}.$$

In the formula Equation (2), *N_ij_* is the number of offspring produced by the breeding pair of the *i*'th male and the *j*'th female. Similarly to model Equation (1), γ are the regression coefficients or effects of the respective variables. All variables have the same meaning as in the logistic model Equation (1) while the estimates coefficients are of course unique for each model.

## 3 RESULTS

### 3.1 Genetic diversity and differentiation

We scored the complete genotypes of 318 adults and 651 offspring at eleven microsatellite markers with no missing genotypes. A total of 193 alleles were scored, and overall total gene diversity, *H*_T,_ was 0.750 (Table 2). Locus SMB101 displayed the highest levels of genetic variability segregating for 40 alleles with *H*_T_ = 0.917 in the total material, while SMA103 only showed four alleles while the lowest total gene diversity was shown at locus SMA11, *H*_T_ = 0.537. Overall, breeders of west origin displayed higher level of genetic variability, both expressed as allelic richness (*A*_r_ = 14.3) and heterozygosity (*H*_E_ = 0.740), than those from the south (*A*_r_ = 11.5 and *H*_E_ = 0.670). Average genetic difference between the two parental groups from different origins (*F*_ST_ = 0.094, *p* < 0.05; Table 3), with single locus pairwise *F*_ST_ estimates ranging from 0.009 at locus SMD110 to 0.240 at locus SMB11 (Table 2), again closely resembling natural conditions (Blanco Gonzalez et al., 2016).

**Table 2:** Summary statistics of genetic variability for the parental groups of south and west origin, offspring, and the total among samples at 11 microsatellite markers

	Locus	SMD121	SMA11	SMA103	SMD131	SMD110	SMD112	SMB11	SMC8	SMB101	SMC5	SMD118	Overall
Breeder south (*n* = 151)	*A*_r_	16.0	5.0	4.0	4.0	10.0	7.0	13.0	12.0	39.0	14.0	14.0	11.5
	*H*_O_	0.850	0.430	0.720	0.480	0.640	0.530	0.490	0.810	0.970	0.770	0.660	0.670
	*H*_E_	0.770	0.550	0.720	0.450	0.650	0.490	0.480	0.750	0.930	0.760	0.790	0.670
	*F*_IS_	−0.106	0.213	0.004	−0.058	0.025	−0.072	−0.012	−0.084	−0.036	−0.015	**0.159**	**0.001**
Breeder west (*n* = 167)	*A*_r_	25.6	4.9	4.0	15.9	9.0	13.9	24.3	13.7	31.4	11.0	15.8	14.3
	*H*_O_	0.890	0.470	0.680	0.740	0.700	0.750	0.830	0.830	0.920	0.810	0.660	0.750
	*H*_E_	0.860	0.450	0.650	0.690	0.650	0.720	0.850	0.810	0.890	0.780	0.830	0.740
	*F*_IS_	−0.029	−0.060	−0.042	−0.069	−0.083	−0.036	0.026	−0.021	−0.028	−0.038	**0.197**	**−0.011**
Offspring (*n* = 651)	*A*_r_	22.2	3.7	4.0	9.1	8.3	11.3	16.5	10.0	19.7	9.7	9.2	11.1
	*H*_O_	0.890	0.550	0.740	0.570	0.630	0.850	0.860	0.840	0.910	0.790	0.730	0.760
	*H*_E_	0.860	0.530	0.680	0.570	0.600	0.780	0.870	0.820	0.890	0.770	0.800	0.740
	*F*_IS_	**−0.030**	−0.039	**−0.076**	−0.003	−0.042	**−0.094**	**0.007**	−0.029	**−0.029**	−0.016	**0.087**	**−0.023**
Total (*n* = 969)	*A*	30	5	4	16	11	14	26	15	40	14	18	193
	*H*_T_	0.879	0.537	0.731	0.591	0.639	0.687	0.819	0.838	0.917	0.780	0.836	0.750
	*F*_IS_	**−0.039**	0.001	**−0.056**	−0.022	−0.037	**−0.080**	0.010	**−0.034**	**−0.028**	−0.018	**0.119**	**−0.016**
	*F*_ST_	**0.060**	**0.057**	**0.068**	**0.032**	0.004	**0.039**	**0.097**	**0.055**	**0.020**	**0.010**	**0.042**	**0.045**
	PIC	0.864	0.471	0.655	0.521	0.549	0.715	0.838	0.812	0.897	0.747	0.801	0.715
	*Q*	0.917	0.429	0.623	0.502	0.518	0.756	0.889	0.847	0.942	0.771	0.839	≈1
	*I*	0.026	0.280	0.139	0.236	0.216	0.092	0.035	0.048	0.017	0.080	0.052	5.5E−13
	fn	−0.004	0.019	−0.010	−0.005	−0.019	−0.033	0.035	−0.002	−0.010	−0.008	0.073	

Note

Bold values indicate significant *p* values at 5% level after the false discovery rate approach (Benjamini & Hochberg, 1995).

Abbreviation: *A*_r_: allelic richness (based on minimum sample size *n* = 151); *F*_IS_: inbreeding coefficient representing deviations from Hardy–Weinberg proportions; fn: frequency of null alleles; *F*_ST_: level of genetic differentiation among all samples;* H*_E_: expected heterozygosity; *H*_O_: observed heterozygosity; *H*_T_: gene diversity (expected heterozygosity) in the total material; *I*: probability of identity index; PIC: polymorphic information content; *Q*: exclusion probability.

**Table 3:** Pairwise *F*_ST_ estimates and corresponding *p* values at 10 microsatellite loci after the false discovery rate correction (Benjamini & Yekutili, 2001)

Sample pair	*F*_ST_	*p* value
Breeder south versus breeder west	0.094	<0.05
Breeder south versus offspring	0.068	<0.05
Breeder west versus offspring	0.012	<0.05

Deviation from HW expectations (*F*_IS_) was observed in 10 of the 3*11 cases generated from the two parental groups and the offspring at 11 loci, with eight of them remaining statistically significant at the 5% level after the FDR correction (Table 2). Four of these eight cases were attributed to excess of heterozygotes in the offspring sample while three of the four cases corresponded to deficiency of heterozygotes at locus SMB118 in both offspring and each of the parental samples. The presence of null alleles at this locus was suggested by CERVUS (estimated null allele frequency = 0.073) and MICROCHECKER analyses (data not shown). Therefore, this locus was only used to resolve parentage assignment for a few “ambiguous” offspring allocated by PAPA (for details, see “Pedigree reconstruction and effective Nb” section under section 22), but it was omitted for further analysis. The other case of heterozygote deficiency was found in the offspring sample at locus SMB11.

Over 30% (41 of 135) of pairwise tests for LD were statistically significant after the FDR correction (at the 5% level, data not shown). Except for the locus pair SMD121‐SMB101 in the breeders from the west coast, all significant pairwise tests were detected in the offspring samples. As deviations from HW and from LD are expected in the heterogenous offspring group, representing a mix of parental stocks and hybrids, no action was taken in response to these findings, except as noted above for locus SMB118.

### 3.2 Pedigree reconstruction and effective number of breeders

**Figure 1:**
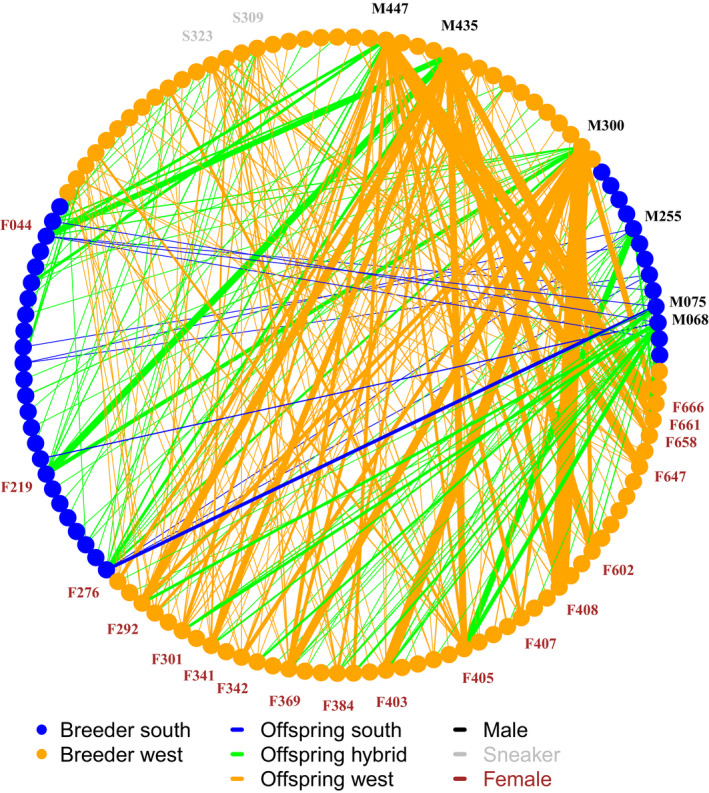
Pedigree reconstruction of 651 offspring collected from the mesocosm basin. Each point of the node represents one parental breeder of south (orange) and west (blue) origin who contributed with at least one offspring to the sample. The lines connecting two nodes represent the offspring of a particular pair or breeders; their color represents whether the offspring were classified as south (blue), west (orange), or hybrid (green); and the line thickness is proportional to the number of offspring comprising each family. The IDs of the nesting male (black), sneaker (gray), and female (red) breeders who contributed to at least 15 offspring are shown in the graph

High PIC and *Q* estimates, and low *I* index, were observed for the set of microsatellite loci (Table 2) and indicating high statistical power to assign true parental pairs to the offspring with this set. Ranking markers based on Excl. P2, PARFEX estimated 100% accumulative success rate of parentage allocation using 11 microsatellites. The pedigree reconstruction of all 651 offspring identified 123 putative breeders out of 318 (i.e., 39%): 71 females and 52 males (Table 4). The total number of parents to the sampled offspring of west origin was 85 (out of a total 167 western individuals released in the mesocosm basin): 22 nesting males, 46 females, and 17 sneaker males. On the other hand, only 38 breeders from south origin (out of 151 released) were found to contribute to the sampled offspring: 13 nesting males and 25 females, with no sneaker male contribution (Table 4). The individuals that contributed the greatest number of offspring were of west origin: males M435, M300, and M447 with 138, 133, and 122 offspring, respectively; and females F405 and F408 with 54 and 50 offspring, respectively (identified by individual IDs along the perimeter of Figure 1). Sneaker male contribution was limited to those of west origin, with S309 and S323 as the main contributors with 16 offspring each. As result, a total of 438 offspring represented 139 families that had both parents of west origin, 195 hybrid offspring represented 93 pairs of different origins, and only 17 offspring represented 11 families with both parents of south origin (Table 4). The largest family identified in this study comprised 31 offspring, and it was assigned to the west origin pair M300‐F403. The parental pair M075‐F276 contributed to the largest family of south origin with just six offspring (Figure 1). Meanwhile, the largest hybrid family comprised 12 offspring from the pair M435‐F219. These large differences in number of offspring resulted in high variance in family sizes (*V*_k_), up to more than 2,000 for western nesting males (Table 5) and, together with skewed contribution of the two sexes, resulted in low inbreeding effective Nb of only 17.6 for the total (west + south) spawning population when sneaker males were not considered. Although none of the sneaker males of south origin contributed to the offspring, the participation of 17 sneaker males from the west coast reduced the variance in family size and resulted in an increase in Nb to 22.9 (Table 5).

**Table 4:** Total number of parents contributing to the offspring (% of total), number of parents contributing to the offspring of south/hybrid/west origin (% of total), total number of offspring (% of total), number of offspring of south/hybrid/west origin (% of origin), number of offspring of south/hybrid/west origin (% by origin), number of families of south/hybrid/west origin (% of total), and largest family size of south/hybrids/west origin (mean ± *SD*)

	South			West		
	Nesting male	Female	Sneaker	Nesting male	Female	Sneaker
Total number of parents	13 (20.3)	25 (32.0)	0	22 (37.3)	46 (54.8)	17 (70.8)
Number of parents per origin	7/11/0 (10.9/17.2/0)	6/23/0 (7.9/30.2/0)	0	0/9/18 (0/15.2/30.5)	0/26/41 (0/30.2/48.8)	0/11/17 (0/47.7/70.8)
Total number of offspring	108 (16.6)	122 (18.7)	0	433 (66.5)	529 (81.2)	110 (16.9)
Number of offspring per origin	17/91/0 (15.7/84.3/0)	17/105/0 (13.9/86.1/0)	0	0/78/355 (0/18.0/82.0)	0/91/438 (0/17.2/82.8)	0/27/83 (0/24.5/75.5)
Number of families per origin	11/43/0 (4.5/17.7/0)	11/50/0 (4.5/20.6/0)	0	0/29/80 (0/11.9/32.9)	0/43/139 (0/17.7/57.2)	0/21/59 (0/8.6/24.3)
Largest family size per origin	6 (0.3 ± 0.9)/11 (1.7 ± 2.1)/0	6 (0.3 ± 0.9)/12 (1.7 ± 2.9)/0	0	0/12 (0.8 ± 1.9)/31 (3.5 ± 5.0)	0/11 (0.5 ± 1.4)/31 (2.4 ± 4.1)	0/3 (0.3 ± 0.6)/1 (2.4 ± 0.9)

**Table 5:** Census of breeders contributing to the offspring (*N*), average number of offspring ($\overset{`}{k}$), variance in reproductive success (*V*_k_), inbreeding effective number of breeders after accounting for variance in family sizes (Nb_f,m_) and also including sex ratio (Nb) for each sample origin separately and for the whole dataset

	Including sneaker males						Excluding sneaker males					
	South		West		All		South		West		All	
	Male	Female	Male	Female	Male	Female	Male	Female	Male	Female	Male	Female
*N*	13	25	39	46	52	71	13	20	22	41	35	61
($\overset{`}{k}$)	8.3	4.9	13.9	11.5	12.5	9.2	8.3	4.8	19.7	10.9	15.5	8.9
*V*_k_	177.9	61.4	1,196.4	215.8	939.3	169.9	177.9	51.7	2,063.8	170.4	1,368.6	138.3
Nb_f,m_	3.7	7.3	5.5	18.0	7.5	24.3	3.7	6.4	3.5	17.4	5.3	26.9
Nb	9.8		16.8		22.9		9.3		11.6		17.6	

Note

Calculations in the left part of the table include families between females and males combining both nesting and sneaker males. Calculations in the right side of the table only consider families formed by nesting males, and families involving sneaker males were excluded in the estimates.

### 3.3 Assortative mating and reproductive fitness

Mating between corkwing wrasse breeders of south and west origin appears to occur randomly with no evidence for assortative mating by population origin (*χ* ^2^ = 0.27, *p* = 0.60 after Yates correction *df* = 1). The logistic regression gave an intercept of the model equaling a basic probability of exp(−5.361)/(1 + exp(−5.361)) = 0.0047 for any random breeding pair to produce at least one offspring. Parameter estimates (in logit) are presented in Table 6. The parameter effects are additive on the response scale, and thus, having a western male in a breeding pair increased the chance of producing offspring to exp(−5.361 + 1.133)/(1 + exp(−5.361 + 1.133)) = 0.0144. Having a breeding pair with both parents from the western population increased the chance of having offspring to exp(−5.361 + 1.133 + 1.083)/(1 + exp(−5.361 + 1.133 + 1.083)) = 0.0412, but since the same pattern was not apparent in breeding pairs of southern origin, the effect of the variable “same origin” was not significant. Parameter estimates (in log) for the analysis of the number of offspring are shown in Table 7. The average number of offspring produced by an average‐sized male mating with an average‐sized female was exp(−2.620 + (0.180*12.806) + (0.055*13.194)) = 1.52. This value increased to exp(−2.620 + (0.180*12.806) + (0.055*13.194)+0.599) = 2.7 if the female was from western origin. On the other hand, neither male origin nor mating between breeders of same origin showed significant increase in offspring production. An average‐sized breeding pair where the male displays sneaking behavior will on average produce exp(−2.620 + (0.180*12.806) + (0.055*13.194) − 0.752) = 0.71 offspring.

**Table 6:** Parameter estimates from the logistic regression model Equation (1) to evaluate the effects on offspring produced of phenotypic traits of the breeders (weight/total length both for males and females), behavior (either nesting or sneaker male), and parental origin (either south or west for both males and females)

Parameter ID	Parameter description	Estimate	*SE*	*p *value
*β*_0_	Intercept	−5.361	0.850	**<0.001**
*β*_1_	Male origin	1.133	0.193	**<0.001**
*β*_2_	Female origin	1.083	0.198	**<0.001**
*β*_3_	Both parents of same origin	−0.167	0.188	0.376
*β*_4_	Male length	−0.026	0.043	0.551
*β*_5_	Female length	−0.029	0.041	0.469
*β*_6_	Sneaker male	0.372	0.144	**0.010**

Note

Bold values represent statistically significant results (*p* < 0.05).

**Table 7:** Parameter estimates from the regression model Equation (2) to evaluate the effects of phenotypic traits of the breeders (weight/total length both for males and females), behavior (either nesting or sneaker male), and parental origin (either south or west for both males and females) on the number of offspring produced by breeding pairs

Parameter ID	Parameter description	Estimate	*SE*	*p *value
*γ*_0_	Intercept	−2.620	0.525	**<0.001**
*γ*_1_	Male origin	0.270	0.156	0.084
*γ*_2_	Female origin	0.599	0.152	**<0.001**
*γ*_3_	Both parents of same origin	0.009	0.144	0.952
*γ*_4_	Male length	0.180	0.027	**<0.001**
*γ*_5_	Female length	0.055	0.024	**0.023**
*γ*_6_	Sneaker male	−0.753	0.119	**<0.001**

Note

Bold values represent statistically significant results (*p* < 0.05).

## 4 DISCUSSION

This study represents one of the few examples evidencing interbreeding between native and translocated wild populations of non‐salmonid marine fish species (Hӓnfling, 2007; Swan et al., 2016). The parental analysis conducted here, in addition to resolving the pedigree of two corkwing wrasse population from south and west Norway, evidenced successful mating between two genetically distinct cleaner fish populations with no evidence for nonrandom mating between them. Thus, there appears to be no intrinsic mechanism against interbreeding and translocated wrasse from the south to salmon hatcheries in western Norway may be expected to mate freely with their western (local) conspecifics. These findings are in line with the recent observations of escaped translocated wrasses in the proximities of salmon farms in the west coast of Norway and putative hybridization with the local population (Faust et al., 2018). We thereby confirm that human‐mediated translocations may facilitate gene flow and break the barriers to interbreeding that are imposed by natural habitat discontinuities on coastal species (Andreakis, Costello, Zanolla, Saunders, & Mata, 2016; Blanco Gonzalez et al., 2016). This reinforces general concerns regarding the admixture of genetically distinct populations (Araki & Schmidt, 2010; Geller et al., 2010; Laikre et al., 2010).

Paternal care marine fish species, such as corkwing wrasse, often display territorial behavior during the spawning season when large nesting males show aggressive behavior to guard their nests against small sneakers (Myhre, Forsgren, & Amundsen, 2012; Potts, 1984; Sinopoli et al., 2018; Stiver et al., 2018). At high population densities, frequent aggressive encounters may interrupt courtship interactions and reduce the occurrence of mating episodes (Myhre et al., 2012). Despite the considerable size of the mesocosm basin (2,000 m^3^), such aggressive encounters between males have likely occurred in the present experimental setting (Halvorsen et al., 2017). Moreover, high population densities may induce higher variance in mating success (Aronsen, Berglund, Mobley, Ratikainen, & Rosenqvist, 2013; Stiver et al., 2018) and alter relative fitness performance (Kokko & Rankin, 2006). The significantly larger contribution of breeders of west origin compared to those from the south (Table 4) suggests putative fitness advantage in western populations, who are typically found at higher densities in nature (Halvorsen et al., 2016). The fact that all breeders employed in this study were wild fish without prior experience in captivity may have offset any disadvantage related to domestication selection (Araki et al., 2009; Christie et al., 2014). The offspring evidenced significant differences in their genetic profile compared to either of the two putative parental populations (Table 4). How these differences may affect their fitness parameters and impact local adaptation is yet to be determined. Although the offspring genotyped here were randomly collected from the mesocosm basin over a 3‐week period, they only represent a portion of the total offspring born in the basin and we cannot dismiss the possibility of some unintentional sampling bias in the offspring. Furthermore, the experimental settings and the absence of information regarding the age of the offspring prevent us from investigating other important factors which have been previously correlated to reproductive fitness, such as timing of spawning or habitat complexity (Blanco Gonzalez et al., 2010; Bose et al., 2018; Moginie & Shima, 2018; Myhre et al., 2012; Sinopoli et al., 2018).

Intraspecific latitudinal variance in life‐history traits generally is strongly correlated to environmental gradients (Munch & Salinas, 2009). Corkwing wrasse populations display strong regional and latitudinal variation in sexual size dimorphism (Halvorsen et al., 2016; Sayer, Gibson, et al., 1996). Populations inhabiting colder areas along the west coast of Norway appeared to present higher proportion of sneaker males, slower growth rates, and delayed maturation (Halvorsen et al., 2016). Despite such differences in life‐history traits, we found no evidence for assortative mating in the mesocosm. The presence of sneaker males had positive effects on offspring production (Table 5 and 6) and the effective Nb (Table 7). These results support previous studies suggesting an important role of sneaker males in sexual size dimorphism and for reproductive success (Perrier, Normandeau, Dionne, Richard, & Bernatchez, 2014; Stiver et al., 2018; Weir, Kindsvater, Young, & Reynolds, 2016). The shorter life span advocated for southern populations (Halvorsen et al., 2016) may have led to high mortalities of wrasses of south origin prior to the spawning event. However, this hypothesis seems unlikely to explain the low contribution of the southern population considering the facts that (a) several breeders of both west and south origin were found alive 2 years after the spawning event took place, when the basin was emptied; and (b) many breeders of south origin only mated with fish from the west coast but not with their southern counterparts (Figure 1).

Our findings provide information on the mating behavior of corkwing wrasse and insight into the putative consequences of intentional large‐scale wild fish translocations on non‐salmonid marine species. We confirmed that translocated wrasses with significantly different phenotypic and genetic profiles to local populations can and do interbreed with local specimens. Faust et al. (2018) suggested that the proportion of translocated‐origin wrasses neighboring a salmon farm in the west coast of Norway may reach almost 40% and our findings provide highly relevant information pertaining to the issue of hybridization and introduction of exogenous genes in such situations. Similarly to corkwing wrasse, intraspecific geographic variance in genetic and phenotypic traits has been also reported on goldsinny and ballan wrasse, the other two main cleaner wrasses used by the salmon industry (Halvorsen et al., 2016; Jansson et al., 2017; Leclercq, Grant, Davie, & Migaud, 2014; Sayer, Gibson, et al., 1996). Hence, considering that millions of cleaner wrasses are annually translocated in Norway (Iversen, 2016) and the UK (Riley et al., 2017) and released inadvertently and intentionally when the net pens are emptied (Blanco Gonzalez & de Boer, 2017), our results should bring awareness of the putative threat pose by wrasse translocations on fitness performance and the long‐term evolutionary potential of recipient populations (Araki, Cooper, & Blouin, 2007; Araki et al., 2009; Eldridge & Naish, 2007; Glover et al., 2017; Laikre et al., 2010).

## CONFLICT OF INTEREST

None declared.

## AUTHOR CONTRIBUTIONS

EBG designed the study, conducted the sampling, and performed the microsatellite analyses. EBG, SHE, and PEJ analyzed the data. EBG wrote the original draft with contribution from all the co‐authors.

## Data Availability

Microsatellite data of every sample genotyped in this study are available in UiT Open Research Data (https://doi.org/10.18710/DSAPAP).
